# An MAGDM method for design concept evaluation based on incomplete information

**DOI:** 10.1371/journal.pone.0277964

**Published:** 2022-11-23

**Authors:** Zhe Chen, Zhao Pan, Qing Ma, Tingting Hou, Peng Zhao

**Affiliations:** 1 Shandong Jiaotong University, Jinan, China; 2 Advanced Manufacturing Technology Research Center, Shandong University of Science and Technology, Qingdao, China; 3 Shijiazhuang Institute of Technology, Shijiazhuang, China; Prince of Songkla University, THAILAND

## Abstract

Design concept evaluation is a huge challenge in the R&D stage of new product development. The information in the assessments often depends on the decision-makers’ individual preferences. However, sometimes the decision-makers cannot give precise and complete information because it is very difficult for them to be familiar with all the criteria. In this situation, an incomplete information decision-making matrix is established. In this paper, decision-making methods based on incomplete information are compared in the literature review. Incomplete information determination method using trust mechanism is proved as a proper way to solve this problem, and the missing information are computed based on the alternatives However, in design concept evaluation, experts commonly provide their preferences using linguistic words according to the different attributes. Hence, we propose a three-step Multiple Attributes Group Decision-making (MAGDM) method where the missing value are determined by attributes. In step one, a data repairing method is proposed based on trust theory. After that, in step two, a comprehensive weight determination method combining AHP and entropy is proposed to obtain the weight of index attributes. Finally, the Rough-TOPSIS method is applied in the design scheme ranking step. In the case study, the proposed method is implemented in a tourism product design process to show its effectiveness.

## 1. Introduction

Design concept evaluation is important in the early stage of new product development (NPD) [1]. Once the design scheme is determined, over 70% of the investment in product design and manufacture is determined [2]. Remodeling the design scheme at a later stage may have a huge adverse impact on the quality, cost, and timeliness of the product [3]. A common method for R&D at the early stage of NPD is to prepare multiple design schemes, which are then judged by the decision-makers (DMs) according to some evaluation attributes, and the decision-making organizer needs to select an optimal alternative according to the information obtained from the decision-makers’ preferences and the design parameters. Later, some improved and modified methods based on the mentioned theories are proposed for specific situations. Problems with several alternatives, which have multiple attributes like this, are usually defined as multiple attribute decision-making (MADM) problems. Hence, design concept evaluation is a critical application of MADM.

How to deal with an imprecise and uncertain environment is a huge challenge in recent studies. To make assessments precise and objective, utility theory, the Analytic Hierarchy Process method (AHP), the graphical method, the Quality Function Deployment method (QFD), and fuzzy logic are applied in design concept evaluation [4]. Due to the uncertainty caused by the subjectivity of the user preferences and for other reasons, fuzzy logic has been integrated in MADM problems for years [5]. Fuzzy logic was first introduced by Zadeh [6], and soon showed its superiority. Its extensions, including interval fuzzy set [7], intuitionistic fuzzy set [8], rough set [9], triangular fuzzy set [10, 11] and trapezoidal fuzzy set [12], are widely applied in related fields including design concept evaluations.

However, most fuzzy logics do not have complete decision-making models, which means the assessment organizers cannot determine the rankings of the alternatives only by the fuzzy set. Instead, fuzzy set normally has a role in building a fuzzy environment to address the uncertainty of the decision-making models. Hence, fuzzy set integrated methods are applied in design concept evaluation and other MADM problems.

The Technique of Order Preference by Similarity to Ideal Solution (TOPSIS), proposed by Hwang and Yoon, is one of the most popular MADM methods, and is widely used in several fields including design concept evaluation [13, 14]. To decrease the subjectivity caused by individuals, the decision organizer recruits more experts in the decision-making process [15], and the MADM methods evaluated by a group of experts are defined as Multiple Attributes Group Decision-making (MAGDM). MAGDM, which integrates the fuzzy set and TOPSIS method, has shown its advantages for design concept evaluation [5]. However, the decision matrix may be incomplete because some experts may not give their opinions quickly based on their backgrounds and experiences [16]. Once the expert group increases in size and the criteria and products become more complicated, more incomplete decision matrices will occur. In previous studies, methods have been implemented to deal with incomplete decision matrices [17, 18]. The trust mechanism is an effective way of determining the missing values. Xu [19] computed the missing data by combining direct and indirect trust. However, in design concept evaluation, experts commonly provide their preferences using linguistic words according to the different attributes, and it is preferred that the missing information is determined by the existing information for the same attribute.

To solve this problem, a novel trust mechanism and Rough-TOPSIS method is proposed in this paper to deal with the uncertain and incomplete information. We first propose a method to determine the missing values relying on the existing information for the corresponding attribute. Meanwhile, we also combine the rough set and the TOPSIS method. The decision matrix is transferred into rough numbers, and a distance based TOPSIS method is applied in the alternative ranking phase. Then a real-life case study is applied for design concept evaluation, and the properties of the improved closeness are analyzed.

The remainder of the paper is structured as follows: Section 2 reviews relevant decision-making models, Section 3 proposes a novel design concept evaluation method based on incomplete information, Section 4 provides an example and some comparisons to demonstrate how the framework can be applied, and Section 5 concludes with some important findings.

## 2. Literature review

This section includes two parts. The related MAGDM studies in design concept evaluation are reviewed in section 2.1, then methods to handle incomplete information are reviewed in section 2.2.

### 2.1 Related MAGDM methods for design concept evaluation

In the R&D of new products, some design concept evaluation methods are proposed as quick decision-making approaches, such as SWOT analysis [20], house of quality [21], Pugh chart [22], and screening matrix [23]. However, because products in modern society are complex, and numerous competing decision factors need to be considered in the development process, MAGDM approaches are implemented in design concept evaluation [15]. There are two main MAGDM approaches. In some MAGDM problems, the decision-makers are asked to give their preferences, and the alternatives are ranked by comparing the preferences without the criteria [24]. This is the alternative-based approach. However, in design concept evaluation, the ranking of the alternatives is relevant to the decision factors (criteria). Hence, it is appropriate to rank the alternatives based on the criteria.

Evaluating the alternatives by distance measurement is one of the most popular theories in MAGDM problems. In distance based ranking methods, the ideal solution is firstly determined by a mathematical model, and the distances between the alternatives and the ideal solution are then calculated. At the end, the alternatives are ranked by the distances. The most common distance based MAGDM methods are the TOPSIS method and the VIKOR method [25, 26].

Enterprises facing tough market competition are paying more attention to user experiences. Under the guidance of user-centered design thinking, expert user groups have participated in the decision-making of solutions, where the participation of the customers largely improves the usability of the products. Meanwhile, the subjectivity of the user preferences results in uncertainty of decision-making information, especially at the design concept evaluation stage. Methods such as the fuzzy set [27] and grey degree theory [28] have been used in MAGDM decision-making. The two methods rely on additional parameters to describe the boundaries of fuzzy intervals, which may increase the subjectivity and complexity of decision-making [29].

The rough set theory, based on the rough number (RN), has shown its advantage in product design decisions. Compared with the other fuzzy methods, the rough set no longer depends on any external information in the data processing process [30]. In other words, this method can only rely on evaluation indicators and objective data for evaluation. Therefore, it can objectively process a large number of subjective data items. In addition, rough sets also show excellent performance in representing the fuzziness of information. In the information processing, the fuzzy numbers are simply expanded the same distance towards both sides. However, the rough set method establishes the interval via a series of rigorous equations. Thus, the rough set method is similar to human cognition behaviors. The evaluation of the design concept is based on human cognition behaviors, the rough set method is suitable for design concept evaluation.

The method describes the uncertainty of decision-making information in the form of rough intervals, transferred from the obtained crisp numbers according to their relationships. Zhai [31] first introduced the concept of rough set of product design evaluation, and proposed a design scheme evaluation method based on the rough set and the gray level. It is difficult to evaluate effectively because of a lack of an evaluation system relying only on the rough set theory. The combination of traditional evaluation system and rough set can achieve better results. In practice, many multiple rough sets based on methods have been successively applied in different product decision-making processes including Rough-TOPSIS [29], Rough-VIKOR [32] and Rough-AHP & Rough-TOPSIS [33]. The TOPSIS based method is considered the commonly used method. Thus, we combine the rough set and TOPSIS method in our research. In design concept evaluations, the experts often give their preferences by linguistic information, which is then transferred into crisp numbers according to the multiple level scale table [34]. In the rough set based on methods, the crisp numbers are then converted into rough numbers.

### 2.2 Handling method for incomplete information in MAGDM

Although we have complete MAGDM methods for design concept evaluation, it is still necessary to develop strategies to deal with an incomplete decision matrix. When the experts make their preferences, it is difficult for them to give accurate judgments on all the criteria due to their lack of knowledge on some criteria. To avoid bias in the raw data, the decision-maker can refuse to give judgment on questions they are not familiar with. Hence, a decision matrix with missing information occurs.

At present, there are some methods for dealing with incomplete information. One way is to determine the optimal alternative without the missing information being completed, where the alternatives are ranked with an incomplete decision matrix. Zhang et al. proposed a two-sided matching decision making (TSMDM) method based on multi-particle hesitant fuzzy linguistic term sets (HFLTS) to simulate this situation, aiming at the problem of TSMDM with high uncertainty, and the matching object may have some hesitancy [35]. The characteristics of the proposed method were verified by taking the matching between the supply and demand of green building technology as an example. Li et al. established the optimization model, and for consensus reaching processes (CRPs) in group decision making (GDM) with incomplete hesitant fuzzy linguistic preference relations (IHFLPRs), proposed some feedback adjustment rules based on local adjustment strategies and an iterative consensus algorithm for GDM with IHFLPRs to ensure the non-randomness and logic of decision-makers. Zhang et al. [36] proposed a new MAGDM method based on multi granulation probabilistic models, Multi-Objective Optimization by Ratio Analysis plus the full multiplicative form and the technique of precise order preference (TPOP) method in incomplete q-rung orthopair fuzzy (q-ROF) information systems was systematically investigated, and the validity of the presented methodology was demonstrated by two experimental studies. Nafei et al. [37] first proposed a modified score function for ranking single-valued neutron sophic numbers. Then, they suggested a TOPSIS method based on the proposed function for decision-making under group recommendation. They provided powerful and practical tools for dealing with uncertainty in decision-making problems. Wang et al. [38] proposed a method based on interval intuitionistic fuzzy numbers. They applied a linear method to fuse each individual intuitionistic fuzzy number (IVIFN), and formed the fused IVIFN of each scheme. The alternatives were then compared and sorted. Wei [39] established an incomplete information processing method by using the traditional grey relational analysis theory (GRA) combined with IVIFS. Liu et al. [40] further proposed an intuitionistic fuzzy multi-attribute decision-making method based on evidence reasoning, converting intuitionistic fuzzy numbers into level of confidence. Finally, the optimal scheme is selected based on the total evaluation value. Hua et al. [17] proposed a DS-AHP method for MAGDM problems with incomplete information. Initially, all possible focal elements directly from the incomplete decision matrix are identified, and then the basic probability assignment of each focal element is determined. Afterwards, the belief intervals of the decision alternatives are evaluated, and preference relations among the alternatives can be computed, and the alternatives are ranked according to the preference relations. Another way of dealing with incomplete information is to estimate the missing values of the incomplete matrix. Hong et al. [41] established a new learning algorithm to repair the missing information based on the rough set theory. Xu et al. [42] integrated the continuous interval argument operator and the continuous interval average operator to transform the incomplete uncertain judgment matrix into a complementary judgment matrix, and proposed a calculation method for the correlation matrix.

Recently, studies of incomplete matrices conducted from the perspective of evaluation experts themselves and their relationships proposed feasible methods for incomplete matrices based on the trust mechanism. Capuano et al. [18] established an incomplete data processing method through expert preference and the trust mechanism, and estimated the missing values through a social influence network model. Xu et al. [19] adopted an incomplete matrix repair method based on the trust mechanism for large-scale and incomplete preference information. Based on the trust mechanism, the direct trust degree and the recommendation trust was calculated, and the incomplete decision matrix was repaired. In other words, the incomplete information can be determined by comparing the trust degree with other decision-makers, and comprehensively calculating the information and trust degree of other decision-makers. It is a practical method to repair missing values, and can meet the needs of common MAGDM methods. The missing values of an expert depend on the information provided by the expert’s most trusted decision-maker. Nevertheless, the trust degree may change among various criteria, meaning that because expert A trusts expert B in terms of a specific attribute does not mean A continues to trust B on another attribute. Hence, it is an appropriate solution to determine the missing data by comparing the trust degree according to the specific attribute.

According to the trust mechanism, the expert confidence can be set as an interval [0, 1], where 0 means without any trust at all, and 1 means with complete trust. Therefore, the trust degree of any expert *x* to expert *y* can be represented by a numerical value in [0, 1]. The overall trust mainly includes direct trust and indirect trust [43], where the direct trust degree represents the mutual trust degree between experts, and the indirect trust degree represents the third party’s trust degree in the expert group. Although the expert groups for design concept evaluation normally consist of professional designers, manufacturers, and experienced customers [15], the selection of decision-making group members has a certain degree of randomness. They are likely to belong to multiple different fields except for their experiences in the specific product. Generally, there is not any direct interaction between decision-making members, and they lack relatively stable trust relationships. Accordingly, in this study, it is believed that the overall trust degree directly depends on the indirect trust degree.

Mostly, the missing values are computed by the existing information. The evaluation mechanism and measurement may be different based on the attributes, but the measurement standards for a specific attribute among the alternatives can be considered to be consistent. Hence, the missing data can be determined based on the attribute.

## 3. Preliminaries

This paper adopts the TOPSIS method and combines rough number theory to evaluate concept design. Although the rough number method has advantages in dealing with uncertain information in the early stage of new product development, it is difficult to form an effective evaluation because it only relies on rough number theory and lacks a relevant evaluation system. Using the TOPSIS combined with rough number theory for evaluation can achieve better results.

The specific process of information treatment is shown as follows [31]:

Let *U* be the universe with the objects according to the crisp numbers. Let object *R* be a set with *n* classes, expressed as *R* = {*C*_1_, *C*_2_, *C*_3_, …, *C*_*n*_}, where *C*_1_ < *C*_2_ < *C*_3_ < ⋯ < *C*_*n*_. Assume *C*_*i*_ is an interval, and *C*_*i*_ ∈ *R*, 1 ≤ *i* ≤ *n*. The lower and the upper limit can be denoted as *C*_*li*_ and *C*_*ui*_, respectively. Then we have *C*_*i*_ = [*C*_*li*_, *C*_*ui*_], where *C*_*li*_ < *C*_*ui*_.[34] ∀ *Y ∈ U*, the lower and the upper approximation can be defined as the equations below.


$$\left\{\begin{array}{c} \underline{Apr}\left(C_{i}\right)=\cup \left\{Y\in U/R\left(Y\right)\leq C_{i}\right\}\\ \bar{Apr}\left(C_{i}\right)=\cup \left\{Y\in U/R\left(Y\right)\geq C_{i}\right\} \end{array}\right.$$ (1)

Where *Apr* (*C*_*i*_) and $\bar{Apr}\left(C_{i}\right)$ illustrate the lower and the upper approximation, respectively. The boundary region of *C*_*i*_ is:

$$Bnd\left(C_{i}\right)=\cup \left\{Y\in U/R\left(Y\right)\neq C_{i}\right\}=\left\{Y\in U/R\left(Y\right)>C_{i}\right\}\cup \left\{Y\in U/R\left(Y\right)<C_{i}\right\}$$ (2)

Let *Lim* (*C*_*i*_) and $\bar{Lim}\left(C_{i}\right)$ be the upper and the lower limit of *C*_*i*_, then

$$\left\{\begin{array}{c} \underline{Lim}\left(C_{i}\right)=\frac{1}{M_{L}}\sum R\left(Y\right)|Y\in \underline{Apr}\left(C_{i}\right)\\ \bar{Lim}\left(C_{i}\right)=\frac{1}{M_{U}}\sum R\left(Y\right)|Y\in \bar{Apr}\left(C_{i}\right) \end{array}\right.$$ (3)

Where *M*_*L*_ / *M*_*U*_ is the number of elements in $\underline{Apr}\left(C_{i}\right)\;/\;\bar{Apr}\left(C_{i}\right)$. The *RN* (*C*_*i*_) can be expressed as:

$$RN\left(C_{i}\right)=\left[\underline{Lim}\left(C_{i}\right),\bar{Lim}\left(C_{i}\right)\right]$$ (4)

The interval of the *RN*(*C*_*i*_) is computed as:

$$RBnd\left(C_{i}\right)=\bar{Lim}\left(C_{i}\right)-\underline{Lim}\left(C_{i}\right)$$ (5)

In a rough set, the *RBnd* (*C*_*i*_) shows the vagueness of the class. The operations of RN are similar to vector operations of intervals, which are shown in Eqs (6)–(9), where *k* represents a constant, *and RN*_1_ and *RN*_2_ represent two RNs.


$$RN_{1}\times k=\left[A_{1},B_{1}\right]\times k=\left[kA_{1},kB_{1}\right]$$ (6)

$$RN_{1}+k=\left[A_{1},B_{1}\right]+k=\left[A_{1}+k,B_{1}+k\right]$$ (7)

$$RN_{1}+RN_{2}=\left[A_{1},B_{1}\right]+\left[A_{2},B_{2}\right]=\left[A_{1}+A_{2},B_{1}+B_{2}\right]$$ (8)

$$RN_{1}\times RN_{2}=\left[A_{1},B_{1}\right]\times \left[A_{2},B_{2}\right]=\left[A_{1}\times A_{2},B_{1}\times B_{2}\right]$$ (9)

## 4. The proposed method based on incomplete information

**Fig 1:**
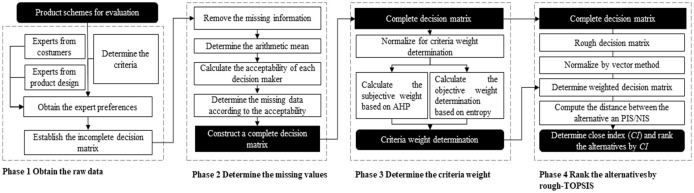
Evaluation model of the cultural tourism product.

This study proposes a decision-making method based on the trust mechanism and the rough-TOPSIS method. A four-phase procedure is proposed in our study as shown in Fig 1. In phase 1, the preparations are made for the assessment, and the incomplete information is obtained from the decision-makers according to the criteria. Then a missing data determination method that integrates other decision-makers and trust degrees is proposed in phase 2. Afterwards, in phase 3 the criteria weights are determined by an integrated method combined with AHP and Shannon entropy. The alternatives are then ranked by a Rough-TOPSIS method.

### 4.1 Obtain the raw data

In the decision preparation phase, the design schemes are prepared as the alternatives, and the criteria need to be chosen and the expert group needs to be arranged. Subsequently, the experts are asked to give their preferences of the alternatives according to the criteria.

Assume *p* decision-makers (*DM*_1_ ~ *DM*_*p*_) are assigned to provide their preferences on *m* design schemes (Alternative 1~ *m*) according to *n* attributes (A_1_~A_n_). The preferences are in the form of a seven-level scale linguistic information shown as Table 1, and the linguistic information is then transferred into crisp numbers.

**Table 1:** Scale of measuring importance and preference and corresponding crisp number.

Linguistic information	Extremely low	Very low	Low	Moderate	High	Very high	Extremely high
	Extremely poor	Very poor	Poor	Neutral	Good	Very good	Extremely good
Crisp number	1	2	3	4	5	6	7

When the decision-maker *DM*_*t*_ (1 ≤ *t* ≤ *p*) is not available to make an accurate judgment on attribute *j* of scheme *i*, the decision-maker can refuse to give the corresponding judgment, then $a_{ij}^{t}$ is recorded as "-", where $a_{ij}^{t}$ represents the judgment made by the *DM*_*t*_ based on scheme *i* and attribute *j*. Related examples are shown in Table 2. To determine the missing values from the raw data and complete all items marked with "-", it is necessary to establish a numerical method to compute the missing information on the basis of existing values.

**Table 2:** The original decision matrix of the multi-attribute evaluation problem.

Attributes	Alternative 1	Alternative 2	…	Alternative m
	*DM*_1_, *DM*_2_, …, *DM*_*p*_	*DM*_1_, *DM*_2_, …, *DM*_*p*_		DM1, DM2, …, DMp
*A*_1_	$a_{11}^{1},\;a_{11}^{2},\;\ldots ,\;a_{11}^{p}$	$a_{21}^{1},\;a_{21}^{2},\;\ldots ,\;a_{21}^{p}$	…	$a_{m1}^{1},\;a_{m1}^{2},\;\ldots ,\;a_{m1}^{p}$
*A*_2_	$a_{12}^{1},\;a_{12}^{2},\;\ldots ,\;a_{12}^{p}$	$a_{22}^{1},\;a_{22}^{2},\;\ldots ,\;a_{22}^{p}$		$a_{m2}^{1},\;a_{m2}^{2},\;\ldots ,\;a_{m2}^{p}$
…	…	…		…
*A*_*n*_	$a_{1n}^{1},\;a_{1n}^{2},\;\ldots ,\;a_{1n}^{p}$	$a_{2n}^{1},\;a_{2n}^{2},\;\ldots ,\;a_{2n}^{p}$		$a_{mn}^{1},\;a_{mn}^{2},\;\ldots ,\;a_{mn}^{p}$

* DM_1_, DM_2_, …, DM_p_ represent the decision-maker 1~p; A1, A2, …, An represent the alternative 1~alternative n

### 4.2 Compute the missing values

Before the assessment, the missing data in the decision matrix need to be calculated. In the proposed method, we use a model by trust mechanism, and missing data are determined by the indirect trust degree. The acquisitions of indirect trust generally come from the evaluation of third-party institutions, or by some calculation methods. In design concept evaluation, the similarity based on method is applied, and the mean of preference values is calculated through the willingness of experts to reach consensus as soon as possible in decision-making problems [44]. In this study, the indirect trust degree of the subjects is judged by the quantitative relationship between each decision data item and the mean value of the preferences. The calculation method is as follows:

The decision matrix *D* of all experts’ judgements for a specific attribute *A*_*j*_ is shown in Table 3.

**Table 3:** Evaluation set of the attribute *A*_*j*_.

	Preference of the attribute *A*_*j*_
Alternative 1	$a_{1j}^{1},\;a_{1j}^{2},\ldots ,a_{1j}^{g},\ldots ,a_{1j}^{g^{\prime }},\ldots a_{1j}^{p}$
Alternative 2	$a_{2j}^{1},\;a_{2j}^{2},\ldots ,a_{2j}^{g},\ldots ,a_{2j}^{g^{\prime }},\ldots a_{2j}^{p}$
…	…
Alternative m	$a_{mj}^{1},\;a_{mj}^{2},\ldots ,a_{mj}^{g},\ldots ,a_{mj}^{g^{\prime }},\ldots a_{mj}^{p}$

Assume *τ* experts “*g*, …, *g*′” in the expert group cannot make accurate judgments on an attribute due to some reasons, where 1 ≤ *g* ≤ *g*′ ≤ *p*, and *τ* ≪ *p*. As is shown in Table 3, each missing information item in the groups $a_{1j}^{g},a_{2j}^{g},\ldots ,a_{mj}^{g}$ to $a_{1j}^{g^{\prime }},a_{2j}^{g^{\prime }},\ldots ,a_{mj}^{g^{\prime }}$ is marked as "-". Hence, the missing values need to be determined according to information provided by other decision-makers.

Let $Arr_{i}=(a_{ij}^{1},a_{ij}^{2},\ldots ,a_{ij}^{p-\tau })$ be the expert decisions after removing ‘$a_{ij}^{g},\ldots ,a_{ij}^{g^{\prime }}$’, where *i* ∈ {1, 2, …, *m*}.

Then the mean value of the preference vector avg_*ij*_ for *Arr*_*i*_ can be expressed by the equation below:

$${\text{avg}}_{ij}=\frac{1}{p-\tau }\sum_{t=1}^{p-\tau }a_{ij}^{t}\;\;\;\;\;\;\;\;,\;\tau \ll p$$ (10)

Where *t* means the sequence of the experts, and *t* ∈ {1, 2, …, *p* − *τ*}. According to Torra and Xu [45, 46], the similarity of expert *t* and avg_*ij*_ can be calculated by Eq (11).


$$\text{sim}\left(a_{ij}^{t},{\text{avg}}_{ij}\right)=1-\frac{\left|a_{ij}^{t}-{\text{avg}}_{ij}\right|}{\sum_{t=1}^{p-\tau }\left|a_{ij}^{t}-{\text{avg}}_{ij}\right|}$$ (11)

Where sim(α, β) means the similarity of α and β. Let $\;acc_{ij}^{t}$ be the acceptability of expert *t*, then

$$acc_{ij}^{t}=\frac{\text{sim}\left(a_{ij}^{t},{\text{avg}}_{ij}\right)}{\sum_{t=1}^{p-\tau }\text{sim}\left(a_{ij}^{t},{\text{avg}}_{ij}\right)}$$ (12)

Considering *m* alternatives of attribute *j* comprehensively, the average acceptability of expert $t\;\bar{acc_{j}^{t}}$ can be computed as:

$$\bar{acc_{j}^{t}}=\frac{1}{m}\sum_{i=1}^{m}acc_{ij}^{t}$$ (13)

The larger the value of $\bar{acc_{j}^{t}}$ is, the easier it is to accept the judgment of expert *t* for attribute *j*. Assume expert *h* has the largest value of $\bar{acc_{j}^{t}}$, the values of $a_{ij}^{g},\ldots ,a_{ij}^{g^{\prime }}$ can be replaced by $a_{ij}^{h}$, thus:

$$\;a_{ij}^{g}=\cdots =a_{ij}^{g^{\prime }}=\;a_{ij}^{h}\;,\;i=1,2,\ldots ,m$$ (14)

Similarly, the other missing data can be determined in the same way, and then we have a complete decision matrix. The decision matrix obtained by expert *t* is shown as:

$$D_{t}=\left[\begin{array}{ccc} a_{11}^{\text{t}} & \cdots & a_{1j}^{t}\\ \vdots & \ddots & \vdots \\ a_{\text{m}1}^{\text{t}} & \cdots & a_{\text{mn}}^{\text{t}} \end{array}\right],\;t=1,2,\ldots ,p$$ (15)

### 4.3 Determine the criteria weight

Criteria weight determination is a vital phase in design concept evaluation, and an integrated weight determination model is established here. In our study, a combination of the subjective weight determination method and the objective method is applied to reserve the decision-makers’ opinion as well as the objective of the assessment [47]. The details of the criteria weight determination are shown, including the subjective weight determination, the objective weight determination and the criteria weight integration.

#### 4.3.1 Calculate the subjective weight based on AHP

The AHP method was first proposed by Saaty [48], and it has become the most important method of the subjective decision-making approach. The core of the method is quantitative analysis by comparing experts’ preferences pairwise. The main steps are as follows:

All the experts need to draw corresponding pairwise conclusions on the relationship between the criteria, using the nine-level scale [48] as shown in Table 4, and transform the experts’ judgment language into the evaluation scale value. We define *u* and *v* as two attributes, and 1 ≤ *u* < *v* ≤ *n*.

**Table 4:** Nine-level scale pairwise comparison of AHP.

Comparison between criteria *u* and *v* (*b*_*uv*_)	Corresponding value
Indifferent between *u* and *v*	1
Weak importance for *u* versus *v*	3
Definite importance for *u* versus *v*	5
Strong importance for *u* versus *v*	7
Very strong importance for *u* versus *v*	9
intermediate value of the judgments	2/4/6/8
Importance of *v* versus *u*	Reciprocal number

Let *b*_*uv*_ be the comparison result of *u* versus *v*, and *b*_*uv*_ is generally obtained from the experts. Hence, a judgment matrix B is established by comparing the relative importance of the evaluation indicators in pairs as follows:

$$B=\left[\begin{array}{cc} \begin{array}{cc} b_{11} & b_{12}\\ b_{21} & b_{22} \end{array} & \begin{array}{cc} \cdots & b_{1n}\\ \cdots & b_{2n} \end{array}\\ \begin{array}{cc} \vdots & \vdots \\ b_{n1} & b_{n2} \end{array} & \begin{array}{cc} & \vdots \\ \cdots & b_{nn} \end{array} \end{array}\right]$$ (16)

Where *b*_*uv*_ = (*b*_*uv*_)^−1^.

The column vector normalization of matrix *B* can be calculated by Eq (17).


$$c_{uv}=\frac{b_{uv}}{\sum_{v=1}^{n}b_{uv}}$$ (17)

Where *c*_*uv*_ is the element of the normalized decision matrix. The row summation of the normalized judgment matrix *C*_*u*_ can be computed by Eq (18).


$$C_{u}=\overset{n}{\underset{v=1}{\sum }}c_{uv}$$ (18)

Thus, the weight of the *u*^th^ row of the normalized decision matrix $w_{j}^{s}$ is calculated as:

$$w_{j}^{s}=\frac{C_{u}}{\sum_{u=1}^{n}C_{u}}$$ (19)

Compute the largest eigenvalue of the decision matrix *λ*_*max*_ using the equation as follows:

$$\lambda_{max}=\frac{1}{n}\overset{n}{\underset{u=1}{\sum }}\frac{{\left(BW\right)}_{u}}{W_{u}}$$ (20)

Where *B* is the judgment matrix and *W* represents the eigenvector $W={\left[w_{1}^{s},w_{2}^{s},\ldots ,w_{n}^{s}\right]}^{T}$. The consistency index of *B* is computed as:

$$C_{I}=\frac{\lambda_{max}-n}{n-1}$$ (21)

Hence, the consistency ratio is computed as the following equation:

$$C_{R}=\frac{C_{I}}{R_{I}}$$ (22)

Where *R*_*I*_ is the random consistency index, and the values of the 1–9 order matrix *R*_*I*_ are shown in Table 5.

**Table 5:** 1~9 order matrix *R*_*I*_ value table.

n	1	2	3	4	5	6	7	8	9
*R*_*I*_	0	0	0.58	0.90	1.12	1.24	1.32	1.41	1.45

When *C*_*R*_ < 0.1, the consistency requirement is met; otherwise, the judgment matrix should be adjusted.

#### 4.3.2 Calculate the objective weight determination based on entropy

The entropy weight method is an important weighting method. In this study, the entropy weight method is used for objective weighting, and the specific weighting method is as follows.

The average value of the experts should be calculated, and the modified decision matrix can be expressed as *D*′, calculated by the equation below:

$$D^{\prime }={\left(\left[r_{ij}\right]\right)}_{m\times n}={\left(\left[\frac{1}{p}\overset{p}{\underset{1}{\sum }}a_{ij}^{\text{t}}\right]\right)}_{m\times n}\;,\;t=1,2,\ldots ,p$$ (23)

According to the entropy weight method, it is necessary to calculate the proportion of the *j*^th^ attribute of the *i*^th^ alternative:

$$P_{ij}=\frac{r_{ij}}{\sum_{i=1}^{m}r_{ij}}$$ (24)

The entropy attribute *j* can be calculated by the following equation:

$$e_{j}=-k\overset{m}{\underset{i=1}{\sum }}P_{ij}\cdot \text{ln}P_{ij}$$ (25)

Where *k* is a constant, and usually *k* = (ln*m*)^−1^. Then the objective weight $W_{j}^{o}$ of judging attribute *j* should satisfy $\sum_{j=1}^{n}W_{j}^{o}=1$, and $W_{j}^{o}\geq 0$. Then

$$w_{j}^{o}=\frac{1-e_{j}}{\Sigma_{j=1}^{n}\left(1-e_{j}\right)}$$ (26)

#### 4.3.3 Integrate criteria weight

The subjective weight mainly depends on the personal subjective tendency of the experts, while the objective weight depends on the numerical characteristics of the assessment data. The integration methods for evaluating data mainly include additive integration and multiplication integration. The integration method is as follows:

$$W_{j}=\frac{{\left(W_{j}^{s}\right)}^{\mu }{\left(W_{j}^{o}\right)}^{\left(1-\mu \right)}}{\sum_{j=1}^{n}{\left(W_{j}^{s}\right)}^{\mu }{\left(W_{j}^{o}\right)}^{\left(1-\mu \right)}}$$ (27)

Where *μ* ∈ [0, 1] is the coefficient showing the important rate of subjective criteria weight. When *μ* > 0.5, the subjective weight outweighs the objective weight, and vice versa.

### 4.4 Rank the alternatives by Rough-TOPSIS

From Eq (15), we define the judgments made on attribute *j* of scheme *i* as:

$$\phi \left(t\right)=\left\{a_{ij}^{1},a_{ij}^{2},\ldots ,a_{ij}^{p}\right\},t=1,2,\ldots ,p$$ (28)

According to rough set theory [29], for any $a_{ij}^{t}$, we have:

$$\left\{\begin{array}{c} U_{ij}^{t-}=\cup \left\{x\in \phi \left(t\right)|\;x\leq a_{ij}^{k}\right\}\\ U_{ij}^{t+}=\cup \left\{x\in \phi \left(t\right)|\;x\geq a_{ij}^{k}\right\} \end{array}\right.$$ (29)

and the upper and lower limit of $a_{ij}^{t}$ can be expressed as:

$$\left\{\begin{array}{c} a_{ij}^{t-}=\bar{y}|\;y\in U_{ij}^{t-}\\ a_{ij}^{t+}=\bar{y}|\;y\in U_{ij}^{t+} \end{array}\right.$$ (30)

Where $a_{ij}^{k-}$ and $a_{ij}^{k+}$ represent the lower and the upper limit of $a_{ij}^{t}$. Let $\bar{y}$ be the arithmetic mean in the corresponding set. So far, for alternative *i* and attribute *j*, the rough set of *a*_*ij*_ can be expressed as a rough number $\left[a_{ij}^{-},a_{ij}^{+}\right]$, where the lower and the upper limits can be calculated by Eq (31):

$$\left\{\begin{array}{c} a_{ij}^{-}=\frac{1}{p}\sum_{t=1}^{p}a_{ij}^{t-}\\ a_{ij}^{+}=\frac{1}{p}\sum_{t=1}^{p}a_{ij}^{t+} \end{array}\right.$$ (31)

In this way, the rough decision matrix *X* can be written as:

$$X=\left[\begin{array}{cc} \begin{array}{cc} \left[a_{11}^{-},a_{11}^{+}\right] & \left[a_{21}^{+},a_{21}^{-}\right]\\ \left[a_{12}^{+},a_{12}^{-}\right] & \left[a_{22}^{+},a_{22}^{-}\right] \end{array} & \begin{array}{cc} \cdots & \left[a_{m1}^{+},a_{m1}^{-}\right]\\ \cdots & \left[a_{m2}^{+},a_{m2}^{-}\right] \end{array}\\ \begin{array}{cc} \vdots & \vdots \\ \left[a_{1n}^{+},a_{1n}^{-}\right] & \left[a_{2n}^{+},a_{2n}^{-}\right] \end{array} & \begin{array}{cc} & \vdots \\ \cdots & \left[a_{mn}^{+},a_{mn}^{-}\right] \end{array} \end{array}\right]$$ (32)

Next, the decision matrix needs to be normalized. According to Chen [49], the normalization processing by the vector method will not change the characteristics of each attribute, which is an ideal solution in TOPSIS. Therefore, the vector method is applied here in this study, and the specific method is shown in Eq (33).


$$\left\{\begin{array}{c} x_{ij}^{-}=\frac{a_{ij}^{-}}{\sqrt{\sum_{i=1}^{m}{\left(a_{ij}^{+}\right)}^{2}}}\\ x_{ij}^{+}=\frac{a_{ij}^{+}}{\sqrt{\sum_{i=1}^{m}{\left(a_{ij}^{+}\right)}^{2}}} \end{array}\right.$$ (33)

Then the normalized decision matrix can be expressed as $X^{\prime }={\left(\left[x_{ij}^{-},\;\;x_{ij}^{+}\right]\right)}_{m\times n}$. The weighted decision matrix is $V={\left(\left[v_{ij}^{-},v_{ij}^{+}\right]\right)}_{m\times n}$, where *$v_{ij}^{-}=W_{j}\times x_{ij}^{-}$* and $v_{ij}^{+}=W_{j}\times x_{ij}^{+}$.

Subsequently, the Positive Ideal Solution (PIS) *v*_*P*_ (*j*) and Negative Ideal Solution (NIS) *v*_*N*_ (*j*) can be determined as:

$$v_{P}\left(j\right)=\left\{\underset{i}{min}\left(v_{ij}^{-}\right),j\in BA;\underset{i}{max}\left(v_{ij}^{+}\right),\;j\in CA\right\}$$ (34)

$$v_{N}\left(j\right)=\left\{\underset{i}{max}\left(v_{ij}^{+}\right),j\in BA\;\underset{i}{min}\left(v_{ij}^{-}\right),\;j\in CA\right\}$$ (35)

Where *BA* and *CA* represent the benefit attribute and the cost attribute, respectively.

Next, let the interval $\left[d_{Pij}^{-},d_{Pij}^{+}\right]$ be the distance between the PIS and $\left[x_{ij}^{-},\;\;x_{ij}^{+}\right]$.


$$\left\{\begin{array}{c} d_{Pij}^{-}=\left\{v_{P}\left(j\right)-v_{ij}^{+},j\in BA;\;v_{ij}^{-}-v_{P}\left(j\right),\;j\in CA\right\}\\ d_{Pij}^{+}=\left\{v_{P}\left(j\right)-v_{ij}^{-},j\in BA;\;v_{ij}^{+}-v_{P}\left(j\right),\;j\in CA\right\} \end{array}\right.$$ (36)

Similarly, the distance from $\left[x_{ij}^{-},\;\;x_{ij}^{+}\right]$ to NIS can be expressed as $\left[d_{Nij}^{-},d_{Nij}^{+}\right]$, shown in Eq (37).


$$\left\{\begin{array}{c} d_{Nij}^{-}=\left\{v_{ij}^{-}-v_{N}\left(j\right),j\in BA;\;v_{N}\left(j\right)-v_{ij}^{+},\;j\in CA\right\}\\ d_{Nij}^{+}=\left\{v_{ij}^{+}-v_{N}\left(j\right),j\in BA;\;v_{N}\left(j\right)-v_{ij}^{-},\;j\in CA\right\} \end{array}\right.$$ (37)

The distance $D_{Pi}=\left[D_{Pi}^{-},D_{Pi}^{+}\right]$ and $D_{Ni}=\left[D_{Ni}^{-},D_{Ni}^{+}\right]$ represent the comprehensive distances from the alternatives to PIS and NIS, respectively. The calculations are shown as Eqs (38) and (39).


$$\left\{\begin{array}{c} D_{Pi}^{-}=\sqrt{\overset{n}{\underset{j=1}{\sum }}{\left(d_{Pij}^{-}\right)}^{2}}\\ D_{Pi}^{+}=\sqrt{\overset{n}{\underset{j=1}{\sum }}{\left(d_{Pij}^{+}\right)}^{2}} \end{array}\right.$$ (38)

$$\left\{\begin{array}{c} D_{Ni}^{-}=\sqrt{\overset{n}{\underset{j=1}{\sum }}{\left(d_{Nij}^{-}\right)}^{2}}\\ D_{Ni}^{+}=\sqrt{\overset{n}{\underset{j=1}{\sum }}{\left(d_{Nij}^{+}\right)}^{2}} \end{array}\right.$$ (39)

According to Song [50], the optimistic coefficient α ∈ [0, 1] can be used to express the optimism for the decision, and the exact distance $D_{Pi}^{*}/D_{Ni}^{*}$ from each scheme to PIS/NIS can be calculated by Eqs (40) and (41). When α is close to 1, it means that the decision-maker is optimistic about the decision result, and when α is close to 0, it means that the decision-maker is not optimistic about the decision result. In general, when α = 0.5, the exact distance is the middle value of the interval.


$$D_{Pi}^{*}=\left(1-\alpha \right)D_{Pi}^{+}+\alpha D_{Pi}^{-}$$ (40)

$$D_{Ni}^{*}=\left(1-\alpha \right)D_{Ni}^{+}+\alpha D_{Ni}^{-}$$ (41)

Then the closeness coefficient *CI* of each scheme is:

$$CI_{i}=\frac{D_{Ni}^{*}}{D_{Pi}^{*}+D_{Ni}^{*}}$$ (42)

The larger the close distance is, the better the solution is and the closer it is to the ideal solution; otherwise, the worse the solution is. The schemes can be ranked through the value of *CIs*.

## 5. Case study

### 5.1 A real-life example: Designing a tourism product

This paper takes the design concept of the ‘Yimeng pancake’ as an example. The pancake is a distinctive food in the area around the Yimeng mountain in China. Recently, a pre-packaged pancake has been developed as a cultural tourism product in this area.

The decision preparation is made in phase 1. Based on the market research, three design concepts of the product are provided by the development team as the alternatives, marked as Alternative 1 to Alternative 3. Fig 1 shows the process of product decision-making.

As shown in Table 6, there are a total of 9 criteria determined from user experience and cultural transmission. A group of 15 designers and 15 customers are invited and assigned to give their preferences to the three design schemes according to the criteria. The linguistic information is then converted into crisp numbers according to Table 1. In this phase, some decision-makers cannot make clear judgments for all criteria, resulting in the incompleteness of the decision matrix. Therefore, the decision matrix needs to be repaired and the missing data items need to be determined.

**Table 6:** Evaluation index system of the product.

Description	Type	Criteria	Category
User acceptance	BA	A1	User experience
Design aesthetics	BA	A2	
Match the fashion tendency	BA	A3	
Development feasibility	BA	A4	
Cost and quality control	BA	A5	
Innovation and competitiveness	BA	A6	
Match the local culture	BA	A7	Cultural transmission
Positive guidance	BA	*A8*	
Cultural heritage	BA	*A9*	

Here we take decision attribute *A5* (cost and quality control) as an example for data patching. As is shown in Table 7, the data for decision-maker 15 is missing.

**Table 7:** Group decision matrix of index attribute A5.

Expert	DM_1_	DM_2_	DM_3_	DM_4_	DM_5_	DM_6_	DM_7_	DM_8_	DM_9_	DM_10_	DM_11_	DM_12_	DM_13_	DM_14_	DM_15_
Alternative 1	6	7	7	7	5	7	6	5	5	4	4	6	6	5	-
Alternative 2	5	4	5	5	4	5	5	5	5	1	3	5	2	5	-
Alternative 3	3	4	3	3	5	3	3	3	5	5	3	6	5	4	-
Expert	DM_16_	DM_17_	DM_18_	DM_19_	DM_20_	DM_21_	DM_22_	DM_23_	DM_24_	DM_25_	DM_26_	DM_27_	DM_28_	DM_29_	DM_30_
Alternative 1	6	5	5	7	6	3	3	4	7	7	7	4	5	4	6
Alternative 2	5	5	5	4	5	7	4	4	6	4	3	4	3	3	6
Alternative 3	5	5	6	3	3	6	5	3	4	3	7	3	3	4	4

After removing the missing values of the decision- maker 15, the decision matrix *D*_*A*5_ is formed. Then, we take the decision attribute *A5* (cost and quality control) as an example. According to Eqs (10)–(13), the average acceptability of decision-making experts $\bar{acc_{A5}^{14}}$ can be determined and the detailed calculation process is as follows:

First, the mean value of the preference vector avg_*iA5*_ (i = 1,2,3) for *Arr*_*i*_ can be expressed by Eq (10):

$${\text{avg}}_{iA5}=\frac{1}{30-1}\sum_{t=1}^{\left(30-1\right)}a_{iA5}^{t}$$ (43)

Then avg_1*A*5_ = 5.48, avg_2*A*5_ = 4.379, avg_3*A*5_ = 4.103

Second, the similarity of experts and avg_*iA*5_ (i = 1,2,3) can be calculated by Eq (11).


$$\text{sim}\left(a_{iA5}^{30-1},{\text{avg}}_{iA5}\right)=1-\frac{\left|a_{iA5}^{30-1}-{\text{avg}}_{iA5}\right|}{\sum_{t=1}^{30-1}\left|a_{iA5}^{t}-{\text{avg}}_{iA5}\right|}$$ (44)

Next, let $\;acc_{ij}^{t}$ be the acceptability of expert *t*, then the acceptability of experts can be calculated as follows:

$$acc_{iA5}^{30-1}=\frac{\text{sim}\left(a_{iA5}^{30-1},{\text{avg}}_{iA5}\right)}{\sum_{t=1}^{30-1}\text{sim}\left(a_{iA5}^{t},{\text{avg}}_{iA5}\right)}$$ (45)

Finally, considering three alternatives of attribute *A*_5_ comprehensively, the average acceptability of experts $\bar{acc_{A5}^{t}}$ can be computed as:

$$\bar{acc_{A5}^{t}}=\frac{1}{3}\sum_{i=1}^{3}acc_{iA5}^{t}$$ (46)

For example, according to the above method, we can calculate the average acceptability of expert 5 of index attribute *A5*. The other detailed calculated values of the average acceptability of decision-making experts of index attribute *A5* are shown in Table 8.

**Table 8:** Average acceptability of decision-making experts of index attribute A5.

Expert	DM_1_	DM_2_	DM_3_	DM_4_	DM_5_	DM_6_	DM_7_	DM_8_	DM_9_	DM_10_
$\bar{acc_{j}^{t}}$	0.0348	0.0349	0.0344	0.0344	0.0350	0.0344	0.0348	0.0348	0.0349	0.0334
Expert	DM_11_	DM_12_	DM_13_	DM_14_	DM_15_	DM_16_	DM_17_	DM_18_	DM_19_	DM_20_
$\bar{acc_{j}^{t}}$	0.0341	0.0345	0.0341	0.0352	-	0.0349	0.0349	0.0345	0.0345	0.0348
Expert	DM_21_	DM_22_	DM_23_	DM_24_	DM_25_	DM_26_	DM_27_	DM_28_	DM_29_	DM_30_
$\bar{acc_{j}^{t}}$	0.0329	0.0343	0.0346	0.0344	0.0345	0.0334	0.0346	0.0345	0.0345	0.0348

As shown in Table 8, $\bar{acc_{A5}^{14}}=0.0352$, which is the maximum value, indicating that the data of decision-maker 14 is most easily accepted by decision-maker 15. Therefore, for the indicator A5, the missing data of decision-maker 15 can be supplemented with reference to decision-maker 14. The decision values for the three alternatives $\left(a_{1A5}^{15},a_{2A5}^{15},a_{3A5}^{15}\right)$ are (5, 5, 4), respectively.

The process of calculating other missing values is the same as above.

In the same way, the entire decision matrix can be completed to form a complete preference matrix *D*′.

Next, determine the criteria weight. The subjective and objective weights of each attribute are obtained by the AHP method and the entropy weight method respectively. When *μ* = 0.5, the weight value of each attribute is shown in Table 9, and the integrated criteria weights *w* are determined.

**Table 9:** Attribute weights of objective indicators of entropy weight method (*α* = 0.5).

	A1	A2	A3	A4	A5	A6	A7	A8	A9
$w_{j}^{s}$	0.062	0.105	0.140	0.063	0.101	0.113	0.128	0.124	0.164
$w_{j}^{o}$	0.100	0.190	0.196	0.097	0.169	0.067	0.081	0.054	0.045
*w*	0.081	0.148	0.168	0.080	0.135	0.090	0.105	0.089	0.105

Then according to Eqs (28)–(32) in step 4, the rough decision matrix *X* can be calculated as shown in Table 10.

**Table 10:** Rough decision matrix *X*.

Alternative\Criteria	A1	A2	A3	A4	A5
Alternative 1	[4.77,5.85]	[4.60,6.15]	[4.49,5.79]	[4.51,5.73]	[4.60,6.30]
Alternative 2	[4.55,5.52]	[4.39,5.29]	[3.71,4.78]	[3.97,5.33]	[3.58,5.15]
Alternative 3	[3.46,4.98]	[2.88,5.00]	[2.97,4.45]	[3.38,4.79]	[3.38,4.90]
Alternative\Criteria	A6	A7	A8	A9	
Alternative 1	[4.89,5.31]	[2.75,3.81]	[2.56,3.79]	[2.57,3.74]	
Alternative 2	[3.72,4.97]	[3.20,4.86]	[2.83,4.54]	[2.71,4.47]	
Alternative 3	[3.59,5.05]	[2.89,4.64]	[2.75,4.55]	[2.84,4.31]	

We take the decision attribute *A5* (cost and quality control) as an example. The detailed calculation process by Eqs (28)–(32) is as follows:

First, from Eq (15), we define the judgments made on attribute *A*_5_ of scheme 1 as:

$$\phi \left(30\right)=\left\{a_{1A5}^{1},a_{1A5}^{2},a_{1A5}^{3},\cdots a_{1A5}^{30}\right\}=\left\{6,6,7,\cdots ,6\right\}$$ (47)

Second, according to rough set theory [29], for $a_{1A5}^{t}$, we have:

$$\left\{\begin{array}{c} U_{1A5}^{t-}=\cup \left\{x\in \phi \left(1\right)|\;x\leq a_{1A5}^{k}\right\}\\ U_{1A5}^{t+}=\cup \left\{x\in \phi \left(1\right)|\;x\geq a_{1A5}^{k}\right\} \end{array}\right.$$ (48)

and the upper and lower limit of $a_{1A5}^{1}$ can be expressed as:

$$\left\{\begin{array}{c} a_{1A5}^{1-}=\{\bar{y}|\;y\leq 6\}\\ a_{1A5}^{1+}=\{\bar{y}|\;y\geq 6\} \end{array}\right.$$ (49)

Next, for alternative 1 and attribute *A*_5_, the rough set of *a*_1*A*5_ can be expressed as a rough number $\left[a_{1A5}^{-},a_{1A5}^{+}\right]$, where the lower and the upper limits can be calculated by Eq (31):

$$\left\{\begin{array}{c} a_{1A5}^{-}=\frac{1}{30-1}\sum_{t=1}^{30-1}a_{1A5}^{1-}=4.9091\\ a_{1A5}^{+}=\frac{1}{30-1}\sum_{t=1}^{30-1}a_{1A5}^{1+}=6.5333 \end{array}\right.$$ (50)

The process of calculating the other values is the same as above, and we can see the other values in Table 10. Finally, the rough decision matrix *X* can be determined.

After normalization, the decision matrix can calculate the distance between each scheme and PIS and NIS and the closeness coefficient *CI* according to Eqs (33)–(42), as shown in Table 11.

**Table 11:** The relative variables and *CIs*.

	$D_{Pi}^{-}$	$D_{Pi}^{+}$	$D_{Ni}^{-}$	$D_{Ni}^{+}$	$D_{Ni}^{+}$	$D_{Ni}^{*}$	*CI*_*i*_
Alternative 1	0.043	0.057	0.046	0.078	0.050	0.062	0.554
Alternative 2	0.042	0.065	0.046	0.068	0.054	0.057	0.515
Alternative 3	0.045	0.083	0.036	0.060	0.064	0.048	0.429

We take the decision attribute *A5* (cost and quality control) in scheme 1 as an example, and the detailed calculation process by Eqs (33)–(42) is as follows:

First, the Positive Ideal Solution (PIS) *v*_*P*_ (*A*_5_) and Negative Ideal Solution (NIS) *v*_*N*_ (*A*_5_) can be determined as:

$$v_{P}\left(A_{5}\right)=\left\{\underset{1}{min}\left(v_{1A_{5}}^{-}\right),A_{5}\in BA;\right\}=0.0767$$ (51)

$$v_{N}\left(A_{5}\right)=\left\{\underset{1}{max}\left(v_{1A_{5}}^{+}\right),A_{5}\in BA;\right\}=0.0411$$ (52)

Where *BA* and *CA* represent the benefit attribute and the cost attribute, respectively.

Next, let the interval $\left[d_{P1A_{5}}^{-},d_{P1A_{5}}^{+}\right]$ be the distance between the PIS and $\left[x_{1A_{5}}^{-},\;\;x_{1A_{5}}^{+}\right]$.


$$\left\{\begin{array}{c} d_{P1A_{5}}^{-}=\left\{v_{P}\left(A_{5}\right)-v_{1A_{5}}^{+},A_{5}\in BA\right\}=0\\ d_{P1A_{5}}^{+}=\left\{v_{P}\left(A_{5}\right)-v_{1A_{5}}^{-},A_{5}\in BA\right\}=0.0207 \end{array}\right.$$ (53)

The distance $D_{P1}=\left[D_{P1}^{-},D_{P1}^{+}\right]$ and $D_{N1}=\left[D_{N1}^{-},D_{N1}^{+}\right]$ represent the comprehensive distances from the alternatives to PIS and NIS, respectively. The calculations are shown as Eqs (38) and (39).


$$\left\{\begin{array}{c} D_{P1}^{-}=\sqrt{\overset{9}{\underset{j=1}{\sum }}{\left(d_{P1j}^{-}\right)}^{2}}=0.0428\\ D_{P1}^{+}=\sqrt{\overset{9}{\underset{j=1}{\sum }}{\left(d_{P1j}^{+}\right)}^{2}}=0.0565 \end{array}\right.$$ (54)

$$\left\{\begin{array}{c} D_{N1}^{-}=\sqrt{\overset{9}{\underset{j=1}{\sum }}{\left(d_{N1j}^{-}\right)}^{2}}=0.0456\\ D_{N1}^{+}=\sqrt{\overset{9}{\underset{j=1}{\sum }}{\left(d_{N1j}^{+}\right)}^{2}}=0.0776 \end{array}\right.$$ (55)

According to Song [50], the optimistic coefficient α ∈ [0, 1] can be used to express the optimism for the decision, and the exact distance $D_{P1}^{*}/D_{N1}^{*}$ from each scheme to PIS/NIS can be calculated by Eqs (40) and (41). When α is close to 1, it means that the decision-maker is optimistic about the decision result, and when α is close to 0, it means that the decision-maker is not optimistic about the decision result. In general, when α = 0.5, the exact distance is the middle value of the interval.


$$D_{P1}^{*}=\left(1-\alpha \right)D_{P1}^{+}+\alpha D_{P1}^{-}=0.0497$$ (56)

$$D_{N1}^{*}=\left(1-\alpha \right)D_{N1}^{+}+\alpha D_{N1}^{-}=0.0616$$ (57)

Then the closeness coefficient *CI* of scheme 1 is:

$$CI_{1}=\frac{D_{N1}^{*}}{D_{P1}^{*}+D_{N1}^{*}}=0.5536$$ (58)

We can calculate the closeness coefficient *CI* of the remaining two schemes according to the above method. The larger the close distance is, the better the solution is and the closer it is to the ideal solution; otherwise, the worse the solution is. The schemes can be ranked through the value of *CIs*.

The process of calculating other values is the same as above. We can see the relative variables and CIs in Table 11.

Let α = 0.5, and it can be seen from the *CIs* that scheme 1 is the best alternative.

### 5.2 Comparison and sensitivity analysis

To reveal the effectiveness of the proposed method, a comparison is conducted. As is shown in Table 12, although all three methods use the trust mechanism for the missing value determination phase, diversities exist among the methods. There are two advantages for design concept evaluation compared with the other methods:

1. The missing values are determined by preferences provided by the experts, and no more information is needed. Compared with the other methods, the proposed method does not need additional information. Considering that the experts are not familiar with each other, it is not possible to obtain the additional information shown in Table 12. Moreover, it is much more objective without additional information in this process. Hence, of the three methods, the proposed method is simple and easy to carry out.
2. The proposed method is based on the criteria, while the others focus on the alternatives. The design concept evaluation is a complicated decision including multiple inconsistent criteria. The criteria are balanced in this process. Thus, multiple criteria are essential for design concept evaluation, and the superiority of the proposed method is obvious.

**Table 12:** Comparison of the three methods for missing value determination.

Method	Capuano [18]	Xu [19]	The proposed method
Missing data generated by	Direct trust	Direct trust & indirect trust	Indirect trust
Determination by	Alternatives	Preferences of alternatives	Preferences of criteria
Input 1	Incomplete rankings	Incomplete alternative preferences	Incomplete criteria preferences
Input 2 (Additional information)	Reliable rankings of the experts	Reliable level of experts (For direct trust only)	None
Steps	1. Input. information 2. Convert fuzzy ranking. 3. Generate social influence network. 4. Calculate missing values.	1. Input. information. 2. Determinate preference vector and arithmetic mean by alternative. 3. Computing acceptability rate. 4. Computing indirect trust degree. 4. Determination direct trust degree 5. Integration direct trust and indirect trust. 6. Determination missing values.	1. Input. information. 2. Determinate preference vector and arithmetic mean by criteria. 3. Computing acceptability rate. 4. Computing indirect trust degree. 5. Determination missing values.

**Fig 2:**
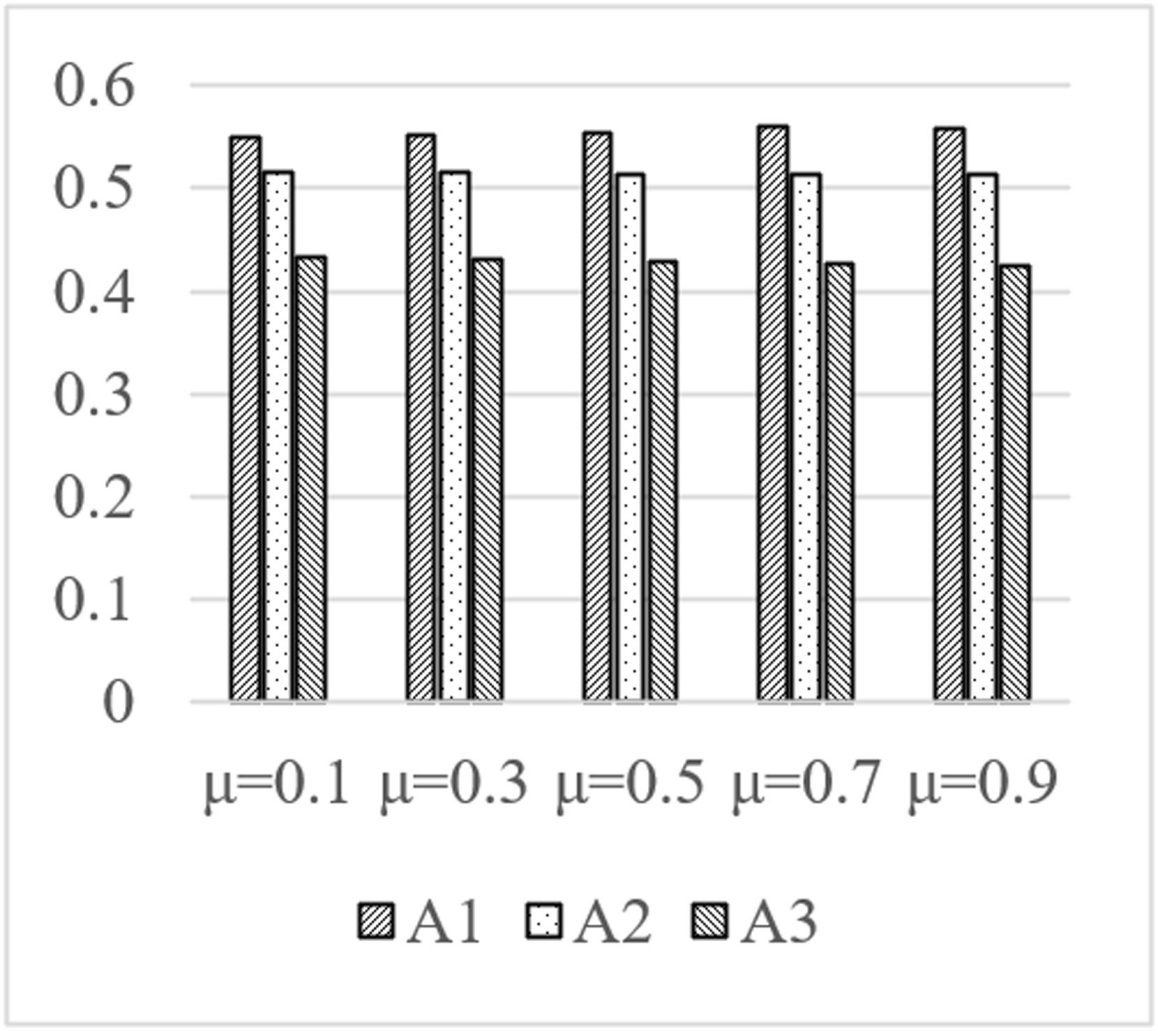
Comparison of closeness coefficients of the three schemes under different evaluation coefficient *μ*.

**Fig 3:**
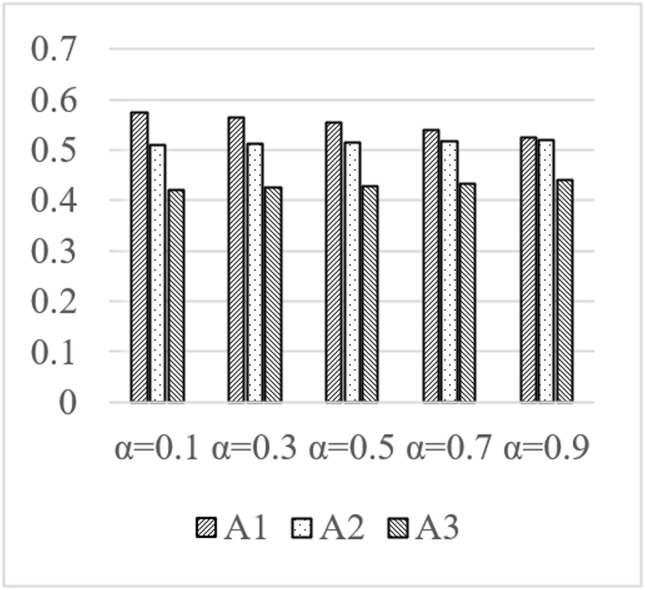
Comparison of closeness coefficients of the three schemes under different evaluation coefficients *α*.

In the design process of real cultural and creative products, the assignment of subjective and objective weights can affect the evaluation results within a certain range. In the proposed method, the weight coefficient *μ* and the optimistic coefficient *α* are the factors that may influence the result. When the value of the coefficient *μ* is 0.1, 0.3, 0.5, 0.7, and 0.9, respectively, the closeness coefficient *CI* of the three schemes is shown in Fig 2. Similarly, when the α equals 0.1 to 0.9, the closeness coefficient *CIs* are shown in Fig 3. Some conclusions are revealed in the figures.

1. When the value of *μ* changes, with the increase of *μ*
  It can be seen from Fig 2 that when the value range of *μ* changes, as the value of the evaluation coefficient *μ* increases, the gap between the three schemes gradually narrows; however, the order of the three schemes has not changed from beginning to end. Scheme A1 is better than the other two alternatives, and its order is A1 > A2 > A3.
2. When the value of *α* changes, with the increase of *α*
  Fig 3 shows that as the value range of modifications and the value of the evaluation optimistic coefficient *α* increases, the gap between the three schemes gradually narrows; however, the order of the three schemes remains constant from beginning to finish. Scheme A1 is superior to the other two options, and its ranking is A1>A2>A3.

## 6. Conclusions

In the design decision-making process of product design options, the source of information is often related to the decision-makers’ personal preferences. Decision-makers are often affected by their own knowledge background, decision-making experience, etc., and sometimes cannot evaluate all decision-making attributes, which will lead to incomplete information in the decision-making matrix. To address the lack of evaluation preference matrix data caused by subjective and objective reasons in product design evaluation, this study proposes a decision-making method for design scheme selection. As an extension of the widely used common decision-making method TOPSIS, this method can be applied to some incomplete information multi-attribute decision-making. The method is applied to the early stage of product design, which can improve the quality and efficiency of design and development. In real life, there are situations where considerable information is missing in the raw data, and *τ* ≪ *p* is not satisfied in Eq (10). In this situation, the proposed method is not accurate and precise because of a lack of raw information. Therefore, how to deal with a large number of missing data values is a challenge in future studies. There are some research gaps in multi-criteria sorting of group decision-making problems (MCS-GDM problems for short). For example, different MCS-GDM problems may have different needs in practical applications. Some MCS-GDM problems may be urgent and need to determine the ranking of alternatives in a short time, while for some nonurgent MCS-GDM problems, experts may want to preserve more initial opinions during the consensus reaching process (CRP). Recognizing these gaps of MCS-GDM problems, and to fill these research gaps, another study proposed an improved Rough-TOPSIS method, which attempts to reduce the imprecision for design concept evaluation in two ways. They applied this method to a numerical example for green building rating and detailed simulation experiments were presented to justify the proposed algorithms [51]. According to social network large-group decision making (SN-LGDM) problems, they aim to investigate the bidirectional interaction-based hybrid consensus strategies of subgroups, and design a minimum adjustment feedback model to help the interacting subgroups pursue greater consensus. In addition, the personalized individual semantic (PIS) model is used to deal with the ambiguity of different semantics of individuals’ expressed opinions [52].

In the future, the following topics can be investigated. First, with the rapid development of information and technology, the interaction of experts can become more and more frequent, and the experts’ comments will be influenced by each other. As a result, it is necessary to introduce new methods to deal with MAGDM problems by considering the consensus reaching process. Second, in some situations, some experts may falsify the final ranking by dishonestly giving their evaluation results. Thus, it is meaningful to study strategic falsifying issues in MAGDM problems. Finally, it will be interesting to further study MAGDM in cases where a large number of experts are involved in problems. Another future research direction is to implement proposal techniques that can be extended to decision problems within the framework of interval valued intuitionistic fuzzy sets as well as interval valued picture fuzzy environments.

## Supporting information

S1 Raw data (PDF)
