# Supplementary material for: An MAGDM method for design concept evaluation based on incomplete information
Source: PLoS One. 2022 Nov 23;17(11):e0277964. doi: 10.1371/journal.pone.0277964 (PMC9683631; doi:10.1371/journal.pone.0277964)
Supplement: S1 Raw data — (PDF) [file pone.0277964.s001.pdf]

Appendix: Pereference values of the alternatives

		DM_1	DM_2	DM_3	DM_4	DM_5	DM_6	DM_7	DM_8	DM_9	DM_{10}	DM_{11}	DM_{12}	DM_{13}	DM_{14}	DM_{15}	DM_{16}	DM_{17}	DM_{18}	DM_{19}	DM_{20}	DM_{21}	DM_{22}	DM_{23}	DM_{24}	DM_{25}	DM_{26}	DM_{27}	DM_{28}	DM_{29}	DM_{30}
Alternative 1	A1	6	7	6	6	5	6	6	5	6	5	5	5	5	4	3	6	5	5	6	7	5	5	5	6	6	6	3	5	5	5
	A2	6	6	7	7	6	7	6	5	6	4	5	4	6	3	6	7	6	5	6	5	6	6	5	7	5	4	3	4	4	5
	A3	5	6	6	6	5	6	5	5	5	5	4	4	7	3	5	7	6	6	5	5	7	5	4	5	5	4	4	4	4	6
	A4	5	6	5	5	5	5	5	5	6	3	5	5	4	5	5	6	6	5	7	6	6	4	4	7	6	5	3	5	4	6
	A5	6	7	7	7	5	7	6	5	5	4	4	6	6	5	-	6	5	5	7	6	3	3	4	7	7	7	4	5	4	6
	A6	5	5	5	5	5	5	5	5	5	5	5	6	5	6	-	6	5	5	5	5	6	4	4	5	5	6	5	5	5	5
	A7	4	4	3	3	3	3	2	2	3	3	3	3	4	4	2	5	3	3	3	5	2	3	3	4	3	2	4	3	4	5
	A8	4	2	3	3	2	3	3	2	3	2	3	3	3	4	2	6	3	3	3	4	2	2	2	3	3	3	4	4	4	6
	A9	4	3	3	3	2	3	3	2	3	2	3	2	5	4	2	5	3	3	3	4	2	3	2	2	3	3	4	4	4	5
Alternative 2	A1	4	5	5	5	5	5	4	4	4	4	4	6	6	6	4	6	5	6	5	6	6	5	4	6	6	6	5	4	5	5
	A2	4	7	5	5	5	5	4	4	4	5	4	5	5	5	5	5	4	5	6	5	6	5	3	5	5	4	5	4	5	6
	A3	4	5	4	4	4	4	4	4	5	3	1	5	4	6	4	5	4	5	4	6	5	4	3	4	4	4	5	4	5	5
	A4	4	4	5	5	4	5	4	4	4	2	4	6	3	6	5	5	5	5	5	6	7	5	3	6	5	5	5	3	5	5
	A5	5	4	5	5	4	5	5	5	5	1	3	5	2	5	-	5	5	5	4	5	7	4	4	6	4	3	4	3	3	6
	A6	4	5	4	4	5	4	5	5	4	2	3	5	5	4	-	6	5	4	4	6	6	5	4	5	3	4	4	2	5	5
	A7	4	3	4	4	4	4	5	5	4	4	2	3	6	4	4	7	4	3	3	4	5	2	1	3	4	5	4	5	5	6
	A8	4	4	4	4	4	4	3	3	3	2	2	3	4	6	4	7	3	2	3	3	5	3	1	2	4	4	5	4	5	5
	A9	4	3	4	4	3	4	3	3	3	1	2	3	5	4	4	6	4	3	3	4	5	2	1	2	4	3	5	5	5	6
Alternative 3	A1	5	5	5	5	5	5	4	3	5	2	2	6	5	6	6	6	4	4	4	3	4	3	3	3	4	5	4	3	4	4
	A2	3	7	3	3	3	3	3	3	3	1	2	5	6	7	5	5	4	4	6	3	1	4	3	4	7	4	4	3	4	4
	A3	4	3	4	4	3	4	3	3	3	1	2	4	4	7	4	5	3	5	4	4	3	4	2	4	4	6	4	2	4	4
	A4	4	5	4	4	4	4	3	3	3	3	3	4	3	6	6	5	5	5	4	4	6	5	3	4	6	2	3	3	4	4
	A5	3	4	3	3	5	3	3	3	5	5	3	6	5	4	-	5	5	6	3	3	6	5	3	4	3	7	3	3	4	4
	A6	3	3	3	3	5	3	3	3	5	5	4	6	6	6	-	6	5	5	3	4	6	6	4	5	3	4	4	4	4	4
	A7	3	4	4	4	5	4	4	3	4	4	2	3	4	5	5	7	5	3	4	4	5	6	1	1	3	2	3	4	3	4
	A8	4	3	4	4	4	4	3	3	3	5	2	3	5	6	5	5	4	2	4	3	5	7	1	1	3	3	3	4	3	3
	A9	4	3	4	4	4	4	3	3	3	5	2	4	3	5	5	6	4	3	4	4	5	4	1	1	3	2	3	4	4	4
